# Seroprevalence of SARS-CoV-2 nucleocapsid antibody among sex workers during the 5th epidemic wave with Omicron variant in Chiang Mai, Thailand

**DOI:** 10.1016/j.heliyon.2024.e36807

**Published:** 2024-08-23

**Authors:** Sayamon Hongjaisee, Ratchadakorn Guntala, Arunrat Tangmunkongvorakul, Nicole Ngo-Giang-Huong, Woottichai Khamduang

**Affiliations:** a Research Institute for Health Sciences, Chiang Mai University, Chiangmai, Thailand; b Maladies Infectieuses et Vecteurs: Écologie, Génétique, Évolution et Contrôle (MIVEGEC), Agropolis University Montpellier, Centre National de la Recherche Scientifique (CNRS), Institut de Recherche Pour le Développement (IRD), Montpellier, France; c LUCENT international collaboration, Faculty of Associated Medical Sciences, Chiang Mai University, Chiangmai, Thailand; d Department of Medical Technology, Faculty of Associated Medical Sciences, Chiang Mai University, Chiangmai, Thailand

**Keywords:** Seroprevalence, SARS-CoV-2 antibodies, Sex workers, COVID-19, Thailand, Anti-nucleocapsid

## Abstract

**Objectives:**

To investigate the seroprevalence of SARS-CoV-2 nucleocapsid antibodies (NC-Ab) in sex workers.

**Methods:**

A cross-sectional/observational study was conducted between March and December 2022 among sex workers living in Chiangmai, Thailand, aged over 18 years and who had engaged in sex work in the previous 12 months. Consenting individuals completed a questionnaire and had blood drawn. IgG-specific for SARS-CoV-2 nucleocapsid was assessed using Euroimmun anti-SARS-CoV-2 NCP ELISA (IgG).

**Results:**

264 sex workers (52.3 % male) with a median age 31 years were included. The overall seroprevalence of SARS-CoV-2 NC-Ab was 42.4 % (44.2 % in males, 40.5 % in females). It was significantly higher among non-Thai than Thai sex workers (57.1 % vs. 37.1 %, *p* = 0.004) and among individuals who reported a history of COVID-19 as compared those who did not (54.9 % vs. 34.3 %, *p* = 0.036). NC-Ab seroprevalence did not differ by sex, age, receipt of COVID-19 vaccines, or the number of vaccine doses. SARS-CoV-2 NC-Ab seropositivity was significantly associated with being non-Thai, having monthly income >15,000 Baht, having received inactivated COVID-19 vaccines, and having been diagnosed with COVID-19.

**Conclusions:**

This study shows a high seroprevalence of NC-Ab among sex workers in Chiangmai, Thailand during the fifth epidemic wave with Omicron variant. This may be due to combined effects of high transmissibility of the Omicron variant and high-risk behavior of those individuals. Specific health education interventions are needed for this specific population.

## Highlights

• Seroprevalence of SARS-CoV-2 NC-Ab IgG was 42.4 % among sex workers in Chiangmai, Thailand.
• NC-Ab IgG seroprevalence was significantly higher among non-Thai than Thai.
• NC-Ab IgG seroprevalence was high among sex workers who reported an history of COVID-19.
• No difference of NC-Ab seroprevalence between unvaccinated and vaccinated sex workers.

## 1 Introduction

Coronavirus disease 2019 (COVID-19), caused by severe acute respiratory syndrome coronavirus type 2 (SARS-CoV-2), first appeared in Thailand in January 2020 [1,2]. The spread of SARS-CoV-2 was successfully controlled with strict measures and closure of borders through 2020. Then, Thailand experienced an increase in the number of COVID-19 cases with the third wave with Alpha variant and a dramatic surge in COVID-19 cases with the fourth wave with Delta variant in 2021 and the fifth wave with Omicron variant in 2022 [[3], [4], [5], [6]]. At the beginning of the Omicron wave (March 2022), it was estimated that about 50 million of people had received 2 doses of vaccine, either an inactivated virus vaccines or viral vector vaccines or mRNA vaccines with a coverage rate of 72 %, one-third had received a third dose (34 %) [7].

Based on the data on COVID-19 Omicron and Delta wave, the pooled proportion of asymptomatic SARS-CoV-2 Omicron infection is 25.5 % [8]; although the actual number of infections is likely to be underestimated, as many asymptomatic persons are never tested and may transmit the infection to others [9,10]. Therefore, the number of reported COVID-19 cases does not fully reflect the COVID-19 outbreak situation. Seroprevalence studies are effective methods for monitoring the spread of infection in a population and for monitoring the pandemic's situation as they detect infection in those who may have had mild or asymptomatic diseases. Anti-nucleocapsid antibodies (NC-Ab) can be used as a marker of SARS-CoV-2 infection in serological surveillance. However, inactivated vaccines can also induce the production of NC-Ab, but the level of NC-Ab declines 3–6 months after vaccination [11] whereas NC-Ab can be detected for several months in unvaccinated individuals who were infected with SARS-CoV-2 naturally [[12], [13], [14]].

Seroprevalence of SARS-CoV-2 antibodies before 2022 was relatively low among people living in Thailand, reflecting the social measures for disease control including national lockdown, curfews, and restriction on travel and movement implemented nationwide in April 2020. Entertainment venues, nightclubs, pubs were also shut down during the COVID-19 pandemic in 2020–2021. In 2021, a study in a cohort of unvaccinated adults in Thailand showed that seroprevalence of SARS-CoV-2 antibodies (anti-S IgG) was 1.4 % [15]. Another study among Thai adults during 2020–2021 showed that NC-Ab IgG was present in 0.4 % of entertainment workers, 1.5–7.5 % of the Bangkok residents and 1.3 % of the Chiang Mai people [16]. Seroprevalence of anti-SARS-CoV-2 antibodies in healthcare providers or hospital staff or individuals at risk due to their occupations varied between 0.2 and 0.9 % depending on the study period [[17], [18], [19]]. A higher seroprevalence 6.5–47.0 % was observed among Thai people returning from high-risk countries [16]. A high seroprevalence 20.5 % was reported in 2021 among household contacts of COVID-19 confirmed cases in Bangkok [20]. SARS-CoV-2 Ab seroprevalence subsequently increased with the spread of the Delta and Omicron variants. A longitudinal serological survey conducted among Thai children during 2021–2022 showed that the infection-induced seropositivity increased from 9.1 % between January and December 2021 to 48.8 % between January and December 2022 [21]. A cross-sectional study conducted in Chonburi, Thailand during October 2022 and January 2023 showed that the number of SARS-CoV-2 infections, as measured by seropositivity of NC-Ab IgG and/or self-reported history of previous infection, was as high as 73.7 % [22].

During the pandemic, sex workers were severely affected by the isolation measures though some continued to sell sex as they have no other incomes. This may contribute to underestimating the spread of virus during the implementation of social measures in the COVID-19 pandemic. Since the beginning of COVID-19 pandemic, various vaccine types have been used in Thailand, including inactivated virus vaccines [CoronaVac (Sinovac) or BBIBP-CorV (Sinopharm)], viral vector vaccines [ChAdOx1-S (AstraZeneca-Oxford)], or mRNA vaccines [BNT162b2 (Pfizer-BioNTech) and mRNA-1273 (Moderna-NIAID)]. However, data on vaccines received by sex workers and SARS-CoV-2 infection in this high-risk group are still lacking. We present herein the seroprevalence of NC-Ab among sex workers in Chiangmai province from March to December 2022.

## 2 Materials and methods

### 2.1 Study population

This study was part of a cross-sectional/observational study conducted from March to December 2022 to investigate the prevalence of sexually transmitted infections among sex workers living in Chiangmai, Thailand. Our study could start in early 2022, after movement restrictions and lockdowns have been gradually lifted. Enrolled participants had to be aged above 18 years and engaged in sex work in the previous 12 months. The study included men or women, Thai, or migrants. At enrollment, data on socio-demographic, health and medical history, potential occupational and non-occupational risks, risk behaviors were collected using an interview questionnaire. Blood samples were collected. Plasma samples were prepared immediately and kept at −70 °C until serological testing was performed.

The study was approved by the Human Experimentation Committee, Research Institute for Health Sciences, Chiang Mai University (Certificate of Ethical approval No.32/2021 and No.40/2022). Written informed consent was obtained from all participants.

### 2.2 Serological assay

Plasma samples collected from consenting participants were tested for IgG-specific for SARS-CoV-2 nucleocapsid (NC) protein with a commercial enzyme linked immunosorbent assay (Euroimmun anti-SARS-CoV-2 NCP ELISA (IgG) immunoassay, Lübeck, Germany) following the manufacturer's instructions. Results were interpreted by calculation of a ratio of the optical density of the control or patient sample over the optical density of the calibrator. Samples with a ratio ≥1.1 were considered IgG positive, and samples with a ratio <1.1 were considered as seronegative.

### 2.3 Statistical analysis

Participants’ characteristics, including sociodemographic and health behavior data, are described in percentages and median, categorical data are presented with percentages and 95 % confidence interval (CI) whereas continuous data are presented with median and interquartile range (IQR). Seroprevalence of NC-Ab was defined as the number of sex workers testing ELISA positive divided by the total number of sex workers tested. The Chi-square test was used to test whether there were any significant differences of seropositivity between each variable. Univariable and multivariable logistic regression analysis was used to determine the potential association between variables and seropositivity. Variables significant at *p* value of <0.250 in the univariable analysis were included in the multivariable logistic regression analysis. Variables that were not statistically significant were removed from the model using a forward selection method. Odd ratio (OR) and 95%CI were calculated to estimate infection-induced seropositivity associated with potential risk factors. All data were analyzed using Stata version 16.0 software (StataCorp, College Station, TX, USA). A *p* value < 0.05 was considered as a statistically significant difference.

## 3 Results

### 3.1 Characteristics of study population

A total of 264 sex workers, median age 31 years (IQR: 25–38 years), of which 47.7 % were female, were included in this study (Table 1). Among these, 194 (73.5 %) are Thai. Two-thirds of participants (68.2 %) were single. About half of them (52.7 %) rented a room, 40.2 % were living alone while 59.8 % were living with their family members. The median of duration of engaging in sex work was 4 years (IQR: 2–8). A majority, 84.8 %, had alcohol drinking habits while 48.9 % had smoking. About 52.2 % of participants (82/157) reported they had been diagnosed with COVID-19. Most participants reported they received COVID-19 vaccine (94.7 %), of which 50.8 % received 2 doses and 40.4 % received 3 doses.

**Table 1:** Characteristics of study population (N = 264).

Characteristics		Total (%)
Sex	Male	138 (52.3)
	Female	126 (47.7)
Median age (years)		31 (IQR: 25–38)
Ethnicity	Thai	194 (73.5)
	Non-Thai	70 (26.5)
Marital status	Single	180 (68.2)
	Has a partner	53 (20.1)
	Separated/Divorced/Widowed	31 (11.7)
Have children		127 (48.1)
Current work	Laborer	162 (61.4)
	Freelance	100 (37.9)
	Business owner	2 (0.7)
Type of accommodation	Renting a room	139 (52.7)
	House	71 (26.9)
	Dormitory	46 (17.4)
	Other	8 (3.0)
Number of family members (persons)	1	106 (40.2)
	2–3	89 (33.7)
	4–5	60 (22.7)
	≥6	9 (3.4)
Median of monthly income (Baht)		15,000 (IQR: 10,000–25,000)
Workplace (can answer >1)	Pub/Bar/Restaurants/Rural road-side bar/Cafe	76 (28.8)
	Traditional massage/Spa/Sauna	73 (27.7)
	Massage parlor	44 (16.7)
	Karaoke	37 (14.0)
	Others	91 (34.5)
Median of duration in sex work (years)		4 (IQR: 2–8)
Smoking		129 (48.9)
Drinking alcohol		224 (84.8)
Drug used, in the past 3 months		55 (20.8)
Self-reported COVID-19 (N = 157)	Yes	82 (52.2)
	No	73 (46.5)
	I don't know	2 (1.3)
Received COVID-19 vaccines	No	14 (5.3)
	Yes	250 (94.7)
Number of COVID-19 vaccine dose	1 dose	15 (6.0)
	2 doses	127 (50.8)
	3 doses	101 (40.4)
	4 doses	7 (2.8)
Type of COVID-19 vaccines	Inactivated + viral vector	84 (33.6)
	Inactivated + mRNA	32 (12.8)
	Inactivated 2 doses	19 (7.6)
	Inactivated 1 dose	6 (2.4)
	Inactivated + viral vector + mRNA	42 (16.8)
	Viral vector 1 dose	2 (0.8)
	Viral vector 2 doses	2 (0.8)
	Viral vector + mRNA	23 (9.2)
	mRNA 1 dose	6 (2.4)
	mRNA 2 or 3 doses	27 (10.8)
	Others	7 (2.8)

### 3.2 Seroprevalence of SARS-CoV-2 NC-Ab

NC-Ab IgG serology was positive for 112 of the 264 tested individuals leading to a prevalence of SARS-CoV-2 infection of 42.4 % (95%CI: 36.6%–48.5 %), Table 2. Seroprevalence was significantly higher among non-Thai than Thai (57.1 % vs. 37.1 %; *p* = 0.004) and among individuals who reported an history of COVID-19 as compared those who did not (54.9 % vs. 34.3 %, *p* = 0.036). In addition, we found that a higher seropositivity was observed in sex workers with a monthly income >15,000 Baht than in those with a monthly income <15,000 Baht (49.6 % vs. 37.1 %, *p* = 0.042).

**Table 2:** Seroprevalence of SARS-CoV-2 NC-Ab among sex workers in Chiangmai, Thailand, 2022.

		N	NC-Ab IgG seropositivity		
			n	% (95%CI)	*p* value
Total		264	112	42.4 (36.6–48.5)	
Sex	Male	138	61	44.2 (36.1–52.6)	0.541
	Female	126	51	40.5 (32.2–49.3)	
Age group	<20 years	13	3	23.1 (7.6–52.3)	0.325
	20–30 years	114	54	47.4 (38.4–56.6)	
	31–40 years	96	38	39.6 (30.3–49.7)	
	41–50 years	41	17	41.5 (27.5–56.9)	
Ethnicity	Thai	194	72	37.1 (30.6–44.2)	**0.004**
	Non-Thai	70	40	57.1 (45.3–68.2)	
Has children	No	137	62	45.3 (37.1–53.7)	0.334
	Yes	127	50	39.4 (31.2–48.1)	
Monthly income (Baht)	<15,000 THB	151	56	37.1 (29.7–45.1)	**0.042**
	>15,000 THB	113	56	49.6 (40.4–58.7)	
Duration in sex work (years)	<2	78	38	48.7 (37.8–59.7)	0.180
	>2	186	74	39.8 (33.0–47.0)	
HIV infection	No	240	103	42.9 (36.8–49.3)	0.609
	Yes	24	9	37.5 (20.7–57.9)	
Smoking	No	135	66	48.9 (40.5–57.3)	**0.030**
	Yes	129	46	35.7 (27.8–44.3)	
Self-reported COVID-19 (N = 155)	No	73	25	34.3 (24.2–45.9)	**0.010**
	Yes	82	45	54.9 (44.0–65.3)	
Received COVID-19 vaccines	No	14	5	35.7 (15.6–62.5)	0.602
	Yes	250	107	42.8 (36.8–49.0)	
COVID-19 vaccines dose (N = 250)	1 dose	15	6	40.0 (19.1–65.3)	0.460
	2 doses	127	50	39.4 (31.2–48.1)	
	3 doses	101	49	48.5 (38.9–58.2)	
	4 doses	7	2	28.6 (7.1–67.5)	
Type of COVID-19 vaccines	Inactivated + viral vector	84	34	40.5 (30.5–51.3)	0.159
	Inactivated + mRNA	32	19	59.4 (41.8–74.8)	
	Inactivated 2 doses	19	11	57.9 (35.5–77.4)	
	Inactivated 1 dose	6	4	66.7 (26.7–91.7)	
	Inactivated + viral vector + mRNA	42	19	45.2 (31.0–60.3)	
	Viral vector 1 dose	2	1	50.0 (5.8–94.2)	
	Viral vector 2 doses	2	1	50.0 (5.8–94.2)	
	Viral vector + mRNA	23	4	17.4 (6.7–38.3)	
	mRNA 1 dose	6	1	16.7 (2.3–63.4)	
	mRNA 2 or 3 doses	27	10	37.0 (21.2–56.3)	
	Others	7	3	42.9 (14.3–77.1)	
	No vaccine received	14	5	35.7 (15.6–62.5)	

NC-Ab seropositivity did not differ by sex (44.2 % in men vs. 40.5 % in women, *p* = 0.541). Among sex workers aged <20 years and 20–30 years, 23.1 % and 47.4 % respectively had antibodies against SARS-CoV-2. The seroprevalence was 39.6 % in sex workers aged 31–40 years and 41.5 % in sex workers aged 41–50 years. The seroprevalence in unvaccinated sex workers (35.7 %), did not differ from the seroprevalence among sex workers who received 1 dose (40.0 %), 2 doses (39.4 %), 3 doses (48.5 %), or 4 doses (28.6 %) of COVID-19 vaccine.

Other variables were also analyzed including marital status, current work, type of accommodation, living alone or with others, workplace, drinking alcohol, or drug use, but no statistically significant difference was found.

### 3.3 Factors associated with SARS-CoV-2 NC-Ab seropositivity

Univariable and multivariable analyses were performed to identify the factors associated with SARS-CoV-2 NC-Ab seropositivity using SARS-CoV-2 NC-Ab serostatus as the dependent variable and participant characteristics as independent variables (Table 3). Although older participants showed a higher seroprevalence than younger ones, this difference was not statistically significant in both univariable and multivariable analyses.

**Table 3:** Factors associated with SARS-CoV-2 NC-Ab seropositivity among sex workers in Chiangmai, Thailand 2022.

Characteristics		Univariable		Multivariable^a^		Multivariable^b^	
		OR (95%CI)	*p*-value	OR (95%CI)	*p*-value	OR (95%CI)	*p*-value
Sex	Male	1.00					
	Female	0.86 (0.53–1.40)	0.541				
Age group	<20 years	1.00					
	20–30 years	3.00 (0.78–11.48)	**0.108**		N.S.		N.S.
	31–40 years	2.18 (0.56–8.45)	0.258				
	41–50 years	2.36 (0.56–9.89)	**0.240**		N.S.		N.S.
Ethnicity	Thai	1.00		1.00		1.00	
	Non-Thai	2.26 (1.30–3.94)	**0.004**	2.51 (1.13–5.57)	**0.024**	2.39 (1.35–4.24)	**0.003**
Have kids	No	1.00					
	Yes	0.79 (0.48–1.28)	0.334				
Type of accommodation	House	1.00					
	Dormitory	1.69 (0.79–3.59)	**0.175**		N.S.		N.S.
	Renting a room	1.48 (0.82–2.67)	**0.192**		N.S.		N.S.
	Others	1.10 (0.24–5.01)	0.898				
Lives with	Alone	1.00					
	With others	1.14 (0.69–1.87)	0.617				
Monthly income (Baht)	≤15,000 THB	1.00		1.00		1.00	
	>15,000 THB	1.67 (1.02–2.73)	**0.043**	2.55 (1.22–5.31)	**0.013**	2.00 (1.19–3.38)	**0.009**
Workplace: Pub/Bar/Restaurants/Rural road-side bar/Cafe	No	1.00					
	Yes	0.72 (0.42–1.25)	**0.244**		N.S.		N.S.
Workplace: Traditional massage/Spa/Sauna	No	1.00					
	Yes	1.72 (0.99–2.96)	**0.051**		N.S.		N.S.
Duration in sex work (years)	<2	1.00					
	>2	0.69 (0.41–1.18)	**0.181**		N.S.		N.S.
Smoking	No	1.00					
	Yes	0.58 (0.35–0.95)	**0.030**		N.S.		N.S.
Self-reported COVID-19 (N = 155)	No	1.00		1.00			
	Yes	2.33 (1.22–4.47)	**0.011**	2.63 (1.28–5.43)	**0.009**		
Received COVID-19 vaccines	No	1.00					
	Yes	1.35 (0.44–4.13)	0.603				
COVID-19 vaccines dose	1 dose	1.00					
	2 doses	0.97 (0.33–2.90)	0.962				
	3 doses	1.41 (0.47–4.26)	0.539				
	4 doses	0.60 (0.09–4.17)	0.605				
Types of COVID-19 vaccine receiving	Non-inactivated vaccines	1.00		1.00		1.00	
	Inactivated vaccines (<6 months)	2.62 (1.25–5.52)	**0.011**	4.05 (0.88–18.58)	0.072	1.87 (1.00–3.51)	**0.050**
	Inactivated vaccines (>6 months)	1.87 (1.01–3.48)	**0.047**	2.51 (1.06–5.91)	**0.036**		N.S.

a Multivariable analysis when using N = 155.

b Multivariable analysis when using N = 264.

Both univariable and multivariate analyses suggested that non-Thai ethnicity was likely to increase the risk of seropositivity compared to Thai ethnicity (adjusted odd ratio, aOR: 2.51, 95 % CI: 1.13–5.57, *p* = 0.024). Participants with a monthly income greater than 15,000 Baht were significantly more likely to be seropositive (aOR: 2.55, 95 % CI: 1.22–5.31, *p* = 0.013). Individuals diagnosed with COVID-19 had a substantially higher risk of seropositivity compared to those who were not diagnosed (aOR: 2.63, 95 % CI: 1.28–5.43, *p* = 0.009). Additionally, those received inactivated COVID-19 vaccines within 6 months before sample collection were more likely to exhibit higher SARS-CoV-2 NC-Ab seropositivity compared to those with no vaccination or vaccinated with viral vector and/or mRNA COVID-19 vaccines (aOR: 4.05, 95 % CI: 0.88–18.58, *p* = 0.072), although this was not statistically significant. Receiving inactivated COVID-19 vaccines more than 6 months prior to sample collection was significantly associated with SARS-CoV-2 NC-Ab seropositivity (aOR: 2.51, 95 % CI: 1.06–5.91, *p* = 0.036).

Due to a significant proportion of participants not reporting their COVID-19 status, only 155 out of 264 individuals were thus included in the model. To maximize the number of participants in the analysis, the variable for self-reported COVID-19 status was omitted from the final model. The analyses demonstrated that ethnicity, monthly income, and type of COVID-19 vaccine were independently associated with a higher risk of SARS-CoV-2 NC-Ab seropositivity. In this study, neither the receipt of COVID-19 vaccines nor the number of vaccine doses significantly influenced serostatus.

## 4 Discussion

In this study, we document a high seroprevalence of SARS-CoV-2 NC-Ab (42.4 %) among sex workers, a high-risk group of SARS-CoV-2 infection, in Chiangmai, Thailand during the fifth epidemic wave with Omicron variant. The seroprevalence of SARS-CoV-2 NC-Ab was significantly higher among non-Thai than Thai sex workers (57.1 % vs. 37.1 %, *p* = 0.004) and among individuals who reported a history of COVID-19 as compared to those who did not (54.9 % vs. 34.3 %, *p* = 0.036). SARS-CoV-2 NC-Ab seropositivity was significantly associated with being non-Thai, having a monthly income >15,000 baht, having been vaccinated with inactivated COVID-19 vaccines, and having been diagnosed with COVID-19. We found no difference in NC-Ab seroprevalence between males and females, consistent with reports from Uganda [23] and Thailand [21,24]. The seroprevalence rate also did not differ by age, receipt of COVID-19 vaccine, or number of vaccine doses.

This finding is consistent with the observation that Omicron variant is more transmissible than other variants of SARS-CoV-2 [25]. Our results are consistent with those of a serological survey conducted among healthy children aged 5–7 years in Thailand which reported that seroprevalence induced by SARS-CoV-2 infection increased from 9.1 % during the pre-Omicron wave (January and December 2021) to 48.8 % during the Omicron wave (January and December 2022) [21]. This high seroprevalence is also consistent with the 73.7 % rate of SARS-CoV-2 infection, defined by positive NC-Ab IgG and/or self-reported history of previous infection during the Omicron wave [22].

Based on a previous study in Denmark 2020, sex workers were 1.9 times more likely to be seropositive compared to non sex workers [26]. This study also demonstrated that being a sex worker or working at a designated safe haven was a significant risk factor of seropositivity compared to those who did not engage in sex work. Another study also showed that sex work is independently associated with higher SARS-CoV-2 seroprevalence among people who inject drugs in the San Diego-Tijuana border region [27]. They also reported that those engaging in sex work were significantly more likely to test seropositive compared to those who did not. In addition, a relatively high prevalence of anti-SARS-CoV-2 antibodies (20.4 %) was observed in adolescent men who have sex with men and transgender women in Brazil [28].

These finding show that sex workers are putting their health at risk during the COVID-19 pandemic. SARS-CoV-2 measures such as social distancing may not be feasible for sex workers since their work require a close contact with their clients and is not possible during sexual transactions. Other behaviors associated with risk of SARS-CoV-2 infection among sex workers include living in the same room with the infected cases, sharing some activities, hugging, holding, sharing objects or unworn masks, similar to household contacts of COVID-19 confirmed cases [20]. Moreover, self-isolation could result in a loss of their income. This suggests that sex workers may have engaged in higher risk behaviors, putting them at greater risk of SARS-CoV-2 infection.

We found that the proportion of non-Thai participants who were seropositive for SARS-CoV-2 infection was higher than that of Thai participants. This may be because the non-Thai participants received more inactivated COVID-19 vaccines whereas the Thai participants received more viral vector and/or mRNA vaccines. This is consistent with previous study showing NC-Ab IgG could be detectable after vaccination with inactivated COVID-19 vaccines [29]. Although we found that higher seropositivity was observed in sex workers with monthly income >15,000 Baht than those with monthly income <15,000 Baht. One hypothesis to explain this is that those with a higher monthly income may have been in contact with more people, or had more jobs, and thus had a higher risk of SARS-CoV-2 infections.

In our study, higher seroprevalence was found among individuals self-reporting COVID-19 than among those who did not. The NC-Ab IgG seropositivity may reflect the true rate of natural infection, including asymptomatic cases or undetected by COVID-19 testing. Our study found that NC-Ab IgG was detected in 45/82 (54.9 %) sex workers with a history of past infection. In addition, NC-Ab IgG was positive in 25/73 (34.3 %) sex workers who reported no history of previous infection, suggesting that they may have been previously infected with SARS-CoV-2 but had an asymptomatic infection and therefore were not tested for COVID-19.

The results also showed that receipt of COVID-19 vaccines or doses of vaccines did not affect the rate of NC-Ab seroprevalence in our population. We also analyzed the different types of COVID-19 vaccines on SARS-CoV-2 antibody seropositivity. Since the beginning of COVID-19 pandemic, various vaccine types have been used in Thailand. We found that sex workers who received inactivated virus vaccines were more likely to be seropositive than those who received viral vector and/or mRNA vaccines. This observation may be explained by the fact that inactivated vaccines can also induce the production of NC-Ab similar to those triggered by natural infection, but NC-Ab decline 3–6 months after vaccination [11,29]. In our population, we found that 57 participants previously received any dose of inactivated vaccines within 6 months before sample collection.

The present study has some limitations. First, this study was not initially designed to investigate SARS-CoV-2, specific information of SARS-CoV-2 infection such as history of infection or clinical manifestation of SARS-CoV-2 infection was not collected. Second, this is a cross-sectional study which does not allow the time of infection to be determined. Third, data on sociodemographic characteristics, sexual behaviors, and health-related characteristics were self-reported. Finally, NC-Ab tends to decrease over time and the detection of NC-Ab may be influenced by the time interval between the infection and blood collection.

## 5 Conclusion

In conclusion, we found that the prevalence of SARS-CoV-2 infection, as measured by NC-Ab IgG seropositivity was high among sex workers in Chiangmai, Thailand indicating the actual number of sex workers infected with Omicron variants during the fifth epidemic wave of the COVID-19 epidemic. The vulnerability of sex worker communities and the results from this study should be considered by public health decision-makers when designing health education or other specific interventions for this population and in preparing for future pandemics.

## Ethical Approval statement

The study was approved by the Human Experimentation Committee, Research Institute for Health Sciences, Chiang Mai University (Certificate of Ethical approval No.32/2021 and No.40/2022).

## Funding

This work was supported by 10.13039/501100002842Chiang Mai University Junior Research Fellowship Program, Thailand.

## Data availability statement

The datasets used and/or analyzed during the current study are available from the corresponding author upon reasonable request.

## CRediT authorship contribution statement

**Sayamon Hongjaisee:** Writing – review & editing, Writing – original draft, Validation, Project administration, Methodology, Investigation, Funding acquisition, Formal analysis, Data curation, Conceptualization. **Ratchadakorn Guntala:** Writing – review & editing, Methodology, Formal analysis. **Arunrat Tangmunkongvorakul:** Writing – review & editing, Resources. **Nicole Ngo-Giang-Huong:** Writing – review & editing, Validation, Investigation, Data curation. **Woottichai Khamduang:** Writing – review & editing, Writing – original draft, Validation, Supervision, Investigation, Data curation, Conceptualization.

## Declaration of competing interest

The authors declare that they have no known competing financial interests or personal relationships that could have appeared to influence the work reported in this paper.
