# Supplementary material for: Seroprevalence of SARS-CoV-2 nucleocapsid antibody among sex workers during the 5th epidemic wave with Omicron variant in Chiang Mai, Thailand
Source: Heliyon. 2024 Aug 23;10(17):e36807. doi: 10.1016/j.heliyon.2024.e36807 (PMC11388775; doi:10.1016/j.heliyon.2024.e36807)
Supplement: Multimedia component 1 [file mmc1.pdf]

Questionnaire survey

Health behavior and prevalence of HIV and Other Infectious Diseases of service workers in sexual entertainment venues in Chiang Mai

Participant ID **F / M -☐☐-☐☐☐**

Interviewer code ....

Date of interview (dd-mm-yy Buddhist Era.) ☐☐-☐☐-☐☐☐☐

Instruction : Information received from the interview will be kept strictly confidential, please be comfortable and answer to these question completely and as accurately as you can. If you have any questions or inquires, you can ask the interviewer any time.

<u>Section 1</u>: Socio-demographic information

1.1 What is your gender?

☐ 1. Male ☐ 2. Female

1.2 How old are you now?

.... Years old.

1.3 Where were you born?

☐ 1. Myanmar

☐ 2. Laos

☐ 3. Thailand

☐ 4. Other (Specify) ______________

1.4 What is your race/ethnicity?

☐ 1. Shan/Tai-Yai ☐ 2. Burmese

☐ 3. Laotian ☐ 4. Cambodian

☐ 5. Chinese ☐ 6. Thai

☐ 7. Other (Specify) ______________

1.5 Have you attended school ?

☐ 1. Yes, for ☐☐ years

☐ 2. No *(if **NO**, skip to **Q1.6**)*

1.5.1 In what education system?

☐ 1.Non-formal education system

☐ 2. Formal education system

1.5.2 What's your educational background?

☐ 1. Primary

☐ 2. Secondary

☐ 3. High school

☐ 4. College/university

☐ 5. Other, (specify) ______________

1.6 What is your religion?

☐ 1. Buddhism ☐ 2. Christianity
☐ 3. Islam ☐ 4. Other (Specify) ______________

1.7 What is your current work place? **(You can choose more than one answer)**

○ 1. karaoke ○ 2. Traditional Massage
○ 3. Spa & Sauna ○ 4. restaurants
○ 5. cafe ○ 6. Rural road-side bar
○ 7. Pub/ Bar ○ 8. Massage Parlor
○ 9. Other (Specify) _________

1.8 What is your current occupation?

☐ 1. Business owner (employee(s))
☐ 2. Freelance (part time)
☐ 3. Labourer / Employed for wages (full time)
☐ 4. Other (Specify) ______________

1.9 How long have you been in Thailand? I've been in Thailand for .... months or ....years

1.10 Do you have ID card for staying in Thailand?

☐ 1. Yes
☐ 2. No *(if **No**, skip to **Q1.11**)*

If yes, type of card is

☐ 1.Passport ☐ 2.Pink card
☐ 3.Non-Thai identification card ☐ 4. Thai National ID Card
☐ 5.Other (Specify) ______________

1.11 Do you have a work permit or document registered in Thailand?

☐ 1.No ☐ 2.Yes
☐ 3. Thai National ID Card

1.12 What health insurance do you have?

☐ 1. Non Thai resident with health insurance ☐ 2. 30-baht health care
☐ 3. Social security ☐ 5. Don't have health insurance/ Self-pay
☐ 6. Don't know/Not sure ☐ 7. Other (Specify) ______________

1.13 What is your marital status?

☐ 1. Single
☐ 2. Have a partner
☐ 3. Separated/ Divorced/ Widowed

1.14 Do you have kid? **(biological kid only)**

☐ 1. Yes, (Specify) ....persons
☐ 2. No

1.15 How many family members do you currently live with? .... persons

1.15.1Accommodation

☐ 1. House ☐ 3. Dormitory
☐ 2. Rent a room ☐ 4. Other, (specify) ....

1.16 How much is your current income? Monthly income______________Baht

1.17 What is the answer below that best describe your current household income when comparing with your household expenses?

☐ 1.Sufficient, with savings
☐ 2.Sufficient, with no savings
☐ 3.Insufficient / not enough to spend

<u>Section 2</u>: Health behavior information

2.1 Normally in **<u>past 3 months</u>**, when you have free time from the job, do you do any vigorous-intensity exercises, sports, fitness or recreational (leisure) activities that cause large increases in breathing or heart rate like [aerobic, running or football,] for at least 10 minutes continuously?

☐ 1. Yes
☐ 2. No *(if **No**, skip to **Q2.2**)*

In a typical week, on how many days do you do vigorous intensity sports, fitness or recreational (leisure) activities? ________ days a week

2.2 Normally in **<u>past 3 months</u>**, when you have free time from the job, do you do any moderate-intensity exercises, sports, fitness or recreational (leisure) activities that causes a small increase in breathing or heart rate such as brisk walking, (cycling, swimming, volleyball) for at least 10 minutes continuously?

☐ 1. Yes
☐ 2. No *(if **No**, skip to **Q2.3**)*

In a typical week, on how many days do you do moderate-intensity sports, fitness or recreational (leisure) activities?________ days a week

2.3 Have you smoked cigarettes during the **past 3 months** ?

☐ 1. Yes

☐ 2. No *(if **No**, skip to **Q2.4**)*

If yes, on average, how many cigarettes per day have you smoked?

☐ 1. Less than 6 cigarettes

☐ 2. 6-10 cigarettes

☐ 3. More than 10 cigarettes

2.4 Have you drunk alcohol during the **past 3 months**?

☐ 1. Yes

☐ 2. No *(if **No,** skip to **Q2.5**)*

If yes, what type of alcohol did you usually drink?

☐ 1.Liqueur ☐ 2.Beer ☐ 3.Wine ☐ 4.Other (Specify) __________

On average, how often did you drink?

☐ 1.Once a month or less ☐ 2.2-3 times a month

☐ 3.Once a week ☐ 4.2-3 times a week

☐ 5.Almost everyday

2.5 **In the past 3 months**, have you used drugs?

☐ 1. Yes, Specify______________

☐ 2. No *(if **No**, skip to **Q2.6**)*

If yes, on average, how often did you use drugs?

☐ 1. Once a month or less ☐ 2. 2-3 times a month

☐ 3. Once a week ☐ 4. 2-3 times a week

☐ 5. Almost everyday

2.6 Have you ever used drug injection?

☐ 1. Yes

☐ 2. No *(if **No**, skip to **Q2.7**)*

If yes, have you ever shared needles with others?

☐ 1. Yes

☐ 2. No

2.7 **In the past 3 months**, have you been sick and have received medical treatment from doctors / health facilities?

☐ 1. Yes, Specify disease(s)/symptom(s) ______________

☐ 2. No *(if **No**, skip to **Q2.8**)*

2.8 Do you have any of the following diseases?

Diseases	No	Yes	Do not know/Do not checkup
1. Hypertension			
2. Chronic renal failure			
3. Diabetes Mellitus			
4. Hypercholesterolemia			
5. Other Disease (Specify) ______			

2.9 Have you ever been diagnosed with………?

Diseases	No	Yes	Do not know /Do not checkup
1. jaundice			
2. liver diseases (hepatitis, cirrhosis, or liver cancer)			

2.10 Has anyone in your **<u>family</u>** ever been diagnosed with liver diseases? (hepatitis, cirrhosis, or liver cancer)

☐ 1. Yes

☐ 2. No *(if **No**, skip to **Q2.11**)*

☐ 3. I don't know *(if **I don't know**, skip to **Q2.11**)*

If yes, have your **<u>family</u>** ever been diagnosed with liver diseases?

(You can choose more than one answer)

O1.1 hepatitis O1.2 cirrhosis O1.3 liver cancer

Relationship (Specify) ______________

2.11 Have you ever been diagnosed with sexually transmitted diseases?

Sexually transmitted disease	Yes /No	Date of diagnosis (MM/YYYY)	Duration of treatment
2.11.1 gonorrhea	☐ 1. Yes<br>☐ 2. No<br>☐ 3. I don't know	__ __/ __ __ __ __	☐ 1. All the time<br>☐ 2. Occasionally<br>☐ 3. Never
2.11.2 Non gonococcal infection	☐ 1. Yes<br>☐ 2. No<br>☐ 3. I don't know	__ __/ __ __ __ __	☐ 1. All the time<br>☐ 2. Occasionally<br>☐ 3. Never
2.11.3 syphilis	☐ 1. Yes<br>☐ 2. No<br>☐ 3. I don't know	__ __/ __ __ __ __	☐ 1. All the time<br>☐ 2. Occasionally<br>☐ 3. Never
2.11.4 Genital Herpes	☐ 1. Yes<br>☐ 2. No<br>☐ 3. I don't know	__ __/ __ __ __ __	☐ 1. All the time<br>☐ 2. Occasionally<br>☐ 3. Never
2.11.5 hepatitis B	☐ 1. Yes<br>☐ 2. No<br>☐ 3. I don't know	__ __/ __ __ __ __	☐ 1. All the time<br>☐ 2. Occasionally<br>☐ 3. Never
2.11.6 hepatitis C	☐ 1. Yes<br>☐ 2. No<br>☐ 3. I don't know	__ __/ __ __ __ __	☐ 1. All the time<br>☐ 2. Occasionally<br>☐ 3. Never
2.11.7 Other (Specify) …….	☐ 1. Yes<br>☐ 2. No<br>☐ 3. I don't know	__ __/ __ __ __ __	☐ 1. All the time<br>☐ 2. Occasionally<br>☐ 3. Never

2.12 Have you ever had an HIV test?

☐ 1. Yes

☐ 2. No *(if **No**, skip to **Q2.13**)*

If **Yes**,

2.12.1 When is the last test ☐☐ years ☐☐ months ago

2.12.2 What is the test result?

☐ 1. Positive

☐ 2. Negative *(if **Negative**, skip to **Q2.13**)*

☐ 3. Don't know *(if **Don't know**, skip to **Q2.13**)*

2.12.3.1 If you are receiving antiretroviral treatment, what is the most recent viral load?

- ☐ 1. Equal 40 copies/ml
- ☐ 2. Less than 40 copies/ml
- ☐ 3. Greater than 40 copies/ml
- ☐ 4. Do not know/ Not sure

2.12.3.2 If you are receiving antiretroviral treatment, what is the most recent CD4?

- ☐ 1. Less than 100 $/mm^3$
- ☐ 2. 100 - 199 $/mm^3$
- ☐ 3. 200-299 $/mm^3$
- ☐ 4. 300- 400 $/mm^3$
- ☐ 4. Greater than 400 $/mm^3$
- ☐ 6. Do not know/ Not sure

2.13 Have you ever had a surgery?

- ☐ 1. Yes (specify the position) ______________
- ☐ 2. No *(if **No**, skip to **Q2.14**)*
- ☐ 3. I don't know *(if **I don't know** , skip to **Q2.14**)*

2.14 Have you ever had a blood transfusion?

☐ 1. No ☐ 2. Yes ☐ 3. I don't know

2.15 Have you ever had needle-stick injuries?

☐ 1. No ☐ 2. Yes ☐ 3. I don't know

2.16 Have you ever had an HBV vaccination?

☐ 1. No ☐ 2. Yes ☐ 3. I don't know

2.17 Have you ever shared sharp personal care items with others?

☐ 1. No ☐ 2. Yes

2.18 Have you ever pierced your ears, nose, or other parts of the body?

☐ 1. No ☐ 2. Yes

2.19 Have you ever had tattoos?

☐ 1. No ☐ 2. Yes

2.20 Have you ever visited a community barber for shaving in males?

☐ 1. No ☐ 2. Yes

2.21 Have you ever had a circumcision in males?

☐ 1. No ☐ 2. Yes ☐ 3. I don't know

Section 3: Sexual behavior

3.1 How would you describe your sexual identity?

☐ 1. Heterosexual ☐ 2. Homosexual
☐ 3. Bisexual ☐ 4.Other (Specify) __________

3.2 How old were you when you had sex for the first time? ....years old.

3.3 Who was your first sexual partner?

☐ 1. Boyfriend/girlfriend ☐ 2. Friend ☐ 3. Acquaintance
☐ 4. Employer ☐ 5. stranger ☐ 6. client
☐ 7. Other (Specify) __________

3.4 Career duration .... months or .... years

3.5 How is your sexual behavior?

	Yes	No
1. Vaginal sex		
2. Anal (insertive)		
3. Anal (receptive)		
4. Oral sex		
5. sex toy		
6. Other (specify) __________		

3.6 How many different people have you had sex with? .... persons

3.7 how many sexual partners have you had sex in the past months?......................persons

3.8 In past months, number of sexual partner per day.................................... persons

3.9 **In the past months**, have you had sex who partner

3. 9.1 husband/wife/lover

☐ 1. Yes
☐ 2. No *(if **No**, skip to **Q3.9.2**)*

IF yes, How often did you or your steady partner use condoms while having sex?

☐ 1. All the time
☐ 2. Occasionally
☐ 3. Never

3.9.2 casual sex (Not sex worker)

☐ 1. Yes

☐ 2. No *(if **No**, skip to **Q3.9.3**)*

IF yes, How often did you or your steady partner use condoms while having sex?

☐ 1. All the time ☐ 2. Occasionally ☐ 3. Never

3.9.3 casual sex (including sex worker)

☐ 1. Yes

☐ 2. No *(if **No**, skip to **Q3.10**)*

IF yes, How often did you or your steady partner use condoms while having sex?

☐ 1. All the time ☐ 2. Occasionally ☐ 3. Never

3.10 When was the **last time** you had sexual intercourse? .... days

3.11 Who was your first sexual partner?

☐ 1. Boyfriend/girlfriend ☐ 2. Friend ☐ 3. Acquaintance

☐ 4. Employer ☐ 5. stranger ☐ 6. client

☐ 7. Other (Specify) __________

3.12 The **last time** you had sexual intercourse, which method did you/your partner use to avoid diseases / pregnancy?

☐ 1. Did not use any method ☐ 2. Withdrawal of penis before ejaculation

☐ 3. Morning after pill ☐ 4. Germ killers

☐ 5. Condom ☐ 6. Oral contraceptive pill

☐ 7. Contraceptive implant ☐ 8. Other (Specify) __________

Section 4: COVID-19

4.1 Have you been vaccinated against COVID-19?

☐ 1. Yes ☐ 2. No

4.2 How many vaccines have you received? Specify .... doses

4.2.1 Vaccine 1 ....Date/Month/Year..................

Which vaccine have you had?

☐ 1. Sinovac ☐ 2. AstraZeneca ☐ 3. Sinopham

☐ 4. Pfizer ☐ 5. Moderna ☐ 6. Other (Specify) ______

4.2.2 Vaccine 2 ....Date/Month/Year..................

Which vaccine have you had?

☐ 1. Sinovac ☐ 2. AstraZeneca ☐ 3. Sinopham

☐ 4. Pfizer ☐ 5. Moderna ☐ 6. Other (Specify) ______

4.2.3 Vaccine 3 ....Date/Month/Year..................

Which vaccine have you had?

☐ 1. Sinovac ☐ 2. AstraZeneca ☐ 3. Sinopham

☐ 4. Pfizer ☐ 5. Moderna ☐ 6. Other (Specify) ______

4.2.4 Vaccine 4 ....Date/Month/Year..................

Which vaccine have you had?

☐ 1. Sinovac ☐ 2. AstraZeneca ☐ 3. Sinopham

☐ 4. Pfizer ☐ 5. Moderna ☐ 6. Other (Specify) ______

4.3 The reason why you are not vaccinated against COVID-19 (choose more than one answer)

☐ 1. Fear of injections or needles

☐ 2. Fear of side effects of vaccines

☐ 3. Fear of death due to injection

☐ 4. Cannot access the vaccines

☐ 5. Have congenital diseases

☐ 6. Family members are not allowed.

☐ 7. Fear of the dangers of vaccines from news or social media.

☐ 8. Others (please specify)

4.4 Have you ever been diagnosed with COVID-19?

☐ 1. Yes ☐ 2. No ☐ 3. I don’t know

Thank you for answering to this questionnaire, the

information from the study will be used to benefit to health service development .
